# Emerging techniques in atherosclerosis imaging

**DOI:** 10.1259/bjr.20180309

**Published:** 2019-10-19

**Authors:** Maaz BJ Syed, Alexander J Fletcher, Rachael O Forsythe, Jakub Kaczynski, David E Newby, Marc R Dweck, Edwin JR van Beek

**Affiliations:** 1 British Heart Foundation Centre of Cardiovascular Science,; 2 Edinburgh Imaging Facility QMRI,

## Abstract

Atherosclerosis is a chronic immunomodulated disease that affects multiple vascular beds and results in a significant worldwide disease burden. Conventional imaging modalities focus on the morphological features of atherosclerotic disease such as the degree of stenosis caused by a lesion. Modern CT, MR and positron emission tomography scanners have seen significant improvements in the rapidity of image acquisition and spatial resolution. This has increased the scope for the clinical application of these modalities. Multimodality imaging can improve cardiovascular risk prediction by informing on the constituency and metabolic processes within the vessel wall. Specific disease processes can be targeted using novel biological tracers and “smart” contrast agents. These approaches have the potential to inform clinicians of the metabolic state of atherosclerotic plaque. This review will provide an overview of current imaging techniques for the imaging of atherosclerosis and how various modalities can provide information that enhances the depiction of basic morphology.

## Introduction

 Atherosclerosis is a chronic immune-modulated pathological process affecting multiple vascular beds and leads to diseases that are a major cause of morbidity and mortality worldwide.^1^ Typically, atherosclerosis has slow progression and a long asymptomatic phase. In the later stages, atherosclerosis may manifest as either exertional symptoms due to luminal narrowing or sudden events related to plaque rupture. The latter may cause local occlusion, as occurs in myocardial infarction. Alternatively, sudden rupture of an atherosclerotic plaque may cause distal embolism. This is typically seen in ischaemic stroke distal to a proximal internal carotid artery plaque rupture.

Structural vascular imaging throughout the body has advanced rapidly. Certain morphological features of atherosclerotic plaques are associated with a higher risk of disease progression.^2^ Combining established imaging strategies with “smart” contrast agents and biological tracers allows us to obtain complementary assessments of biological activity within plaques. This carries the potential to improve our pathophysiological understanding, leading to better patient risk stratification and management.

This review will focus on the techniques available to image atherosclerotic disease. We will describe how certain modalities are well suited to image specific cardiovascular components. Finally, we will illustrate how studying biological activity within plaque advances our ability to detect disease, assess risk and guide patient management.

## Biology of atherosclerosis

**Figure 1.**
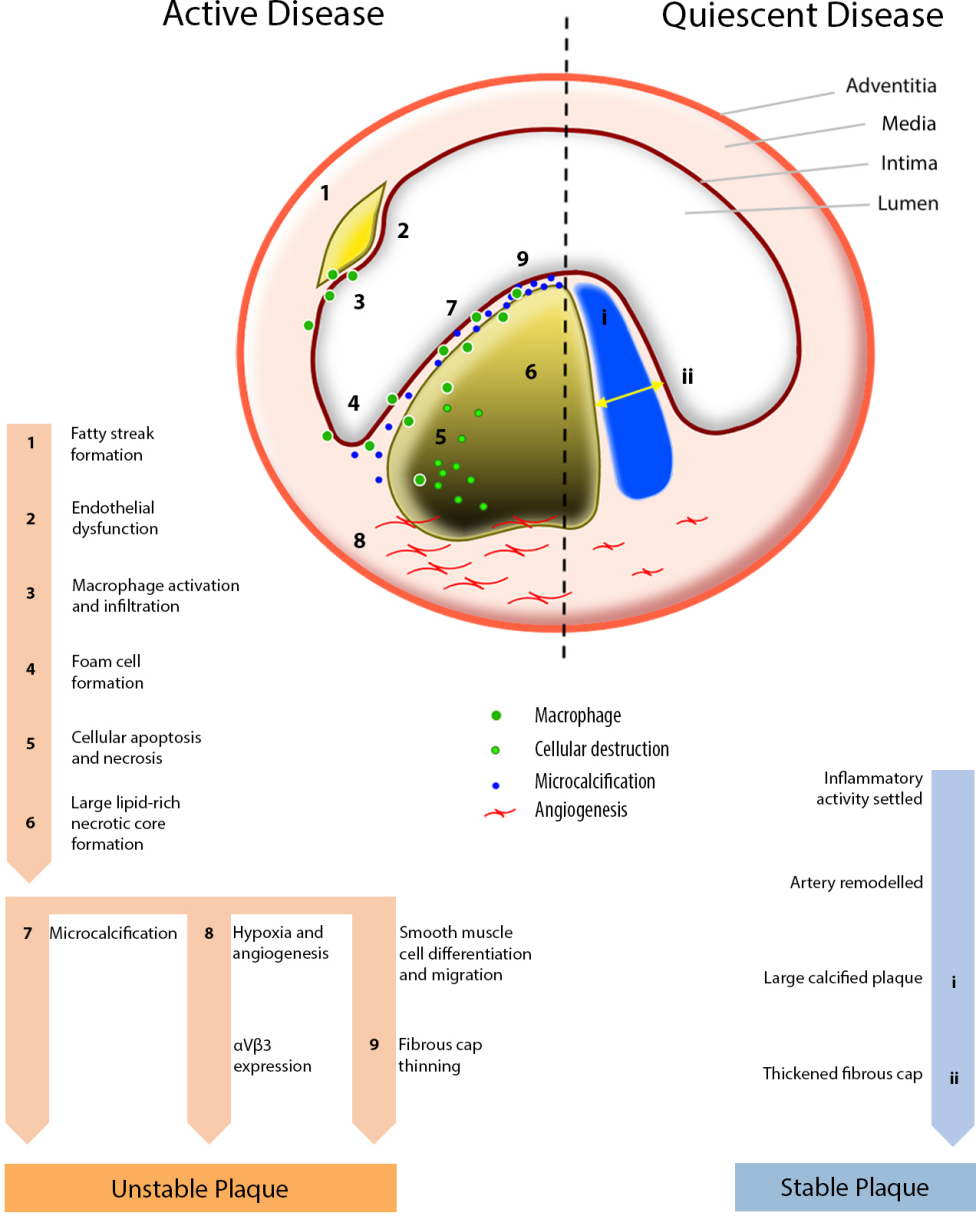
Pathophysiology of atherosclerosis. Arterial cross-section showing that active atherosclerotic disease is characterized by intense biological activity resulting from macrophage infiltration in response to the subendothelial accumulation of oxidized lipoproteins. A cascade of events leads to cell death and the formation of a lipid-rich necrotic core. Localized hypoxia from the lipid-rich core promotes α_v_β_3_ expression and angiogenesis. Thinning of the fibrous cap results from macrophage infiltration and loss of vascular smooth muscle cells. Cell death around the necrotic core leads to microcalcification. This biologically active plaque is at high risk of rupture. In contrast, quiescent atherosclerotic disease represents chronic healed inflammation with positive remodelling. Calcification of the fibrous cap adds stability. This quiescent plaque is at low risk of rupture

Atherosclerosis is a multifocal immunoinflammatory condition of medium and large-sized arteries.^3^ Exposure to established risk factors, such as hypertension, hypercholesterolaemia and smoking, creates a systemic environment that encourages endothelial dysfunction,^4^ oxidation of lipoproteins,^5^ production of free oxygen radicals^6^ and leukocyte migration.^7^ Accumulation of oxidized lipoproteins within the vessel wall forms fatty streaks. Macrophages migrate across the endothelium to phagocytose these lipid-rich proteins. The high intracellular cholesterol content of macrophages induces pathways of cell death. The resultant debris, along with necrotic endothelial and smooth muscle cells, forms the principal constituent of the lipid-rich core (Figure 1).^8^

Atherosclerotic plaques consist of necrotic debris contained by outward remodelling and a fibrous cap within the intimal surface. Vulnerable plaques are prone to rupture and have pathoanatomical features, such as large lipid-rich necrotic cores contained by a thin (<65 µm) macrophage-rich fibrous cap and microcalcification.^9^ In metabolically active plaques, macrophages accumulate in the fibrous cap and degrade the extracellular matrix that is produced by vascular smooth muscle cells.^7^ The necrotic core creates a microenvironment of hypoxia and stimulates angiogenesis.^10^

Calcium deposition in vessel wall inflammation occurs as a macrophage-mediated reparative response to oxidized lipid deposition and endothelial dysfunction. However, rather than being a passive by-product of degradation, the process of vascular calcification is active and controlled. Calcification in atherosclerosis primarily affects the intima. This is in contrast to medial calcification typically observed in patients with diabetes or chronic kidney disease, which adopts a concentric transmural morphology.^11^ The initiation of vascular calcification occurs on a microscopic level (beyond the resolution of conventional imaging) and involves the deposition of calcium and phosphate-rich hydroxyapatite crystals.^12^ This early stage of “microcalcification” signifies intense biological activity and is associated with increased plaque vulnerability. In contrast, the larger established deposits of macroscopic calcification that ultimately develop are associated with plaque stability and a more quiescent phase of disease.

Vulnerable plaques have a thin fibrous cap that is devoid of vascular smooth muscle cells and exhibits intense macrophage recruitment.^13^ Rupture of the thin fibrous cap exposes the lipid-rich necrotic core to luminal flowing blood, initiating rapid and aggressive thrombosis that can cause vascular occlusion. However, plaque rupture is frequently silent and subclinical. Over time, vascular remodelling incorporates the exposed thrombus and the degree of stenosis may worsen.

Angiogenesis and collateralization can partially compensate for reduced blood flow caused by increasingly stenotic vessels. However, if arterial disruption is rapid, this compensatory angiogenesis has had insufficient time to occur, and the resultant ischaemia is more severe. Thus, vulnerable plaque rupture may cause sudden arterial occlusion, loss of tissue perfusion and catastrophic end-organ infarction. Prompt therapeutic reperfusion is required to minimize irreversible tissue loss in a highly time-dependent process.^14^

## Anatomical imaging

Anatomical imaging is diverse and flexible. The chief objectives are to detect luminal narrowing and characterize atherosclerotic plaque. Imaging also allows a global assessment of atherosclerotic disease burden within the entire vascular territory. This information helps clinicians stratify the risk of future adverse events.

Catheter-based contrast angiography is the most frequently used method to image the coronary vessels. This is largely because of the high spatial and temporal resolution that can be obtained. Indeed, catheter angiography is also a platform for intervention. However, catheter angiography alone merely provides a “lumenogram” of the coronary circulation. Plaque characterization is possible but requires specialist equipment. Intravascular optical and ultrasound imaging allow the constituents of plaque morphology to be assessed in remarkable detail. These techniques are an excellent choice to image high-risk patients who may require simultaneous intervention.

There is a need to identify adverse plaque features using non-invasive imaging techniques. This is especially true in low- and medium-risk patient cohorts. Modern cardiovascular medicine has seen increased adoption of non-invasive imaging techniques. Angiography by CT or MRI is safe and offers a reliable alternative to catheter angiography. These imaging modalities can simultaneously image the vessel wall and any surrounding atherosclerosis. Similarly, ultrasound can determine blood flow across a lesion to quantify stenosis. Spectral analysis and specialized contrast agents can characterize plaque at the same time. Non-invasive imaging may also improve clinical decision making. It separates diagnosis from intervention, thus allowing rational decision making by a multidisciplinary team.

Here we discuss the imaging techniques available to visualize morphological features of atherosclerotic vessels. We touch briefly on how these techniques can help characterize plaque.

### Intravascular imaging

Intravascular imaging techniques have the benefit of capturing images from close proximity to the plaque, thus offering extremely high-resolution images.

#### Optical coherence tomography

**Figure 2.**
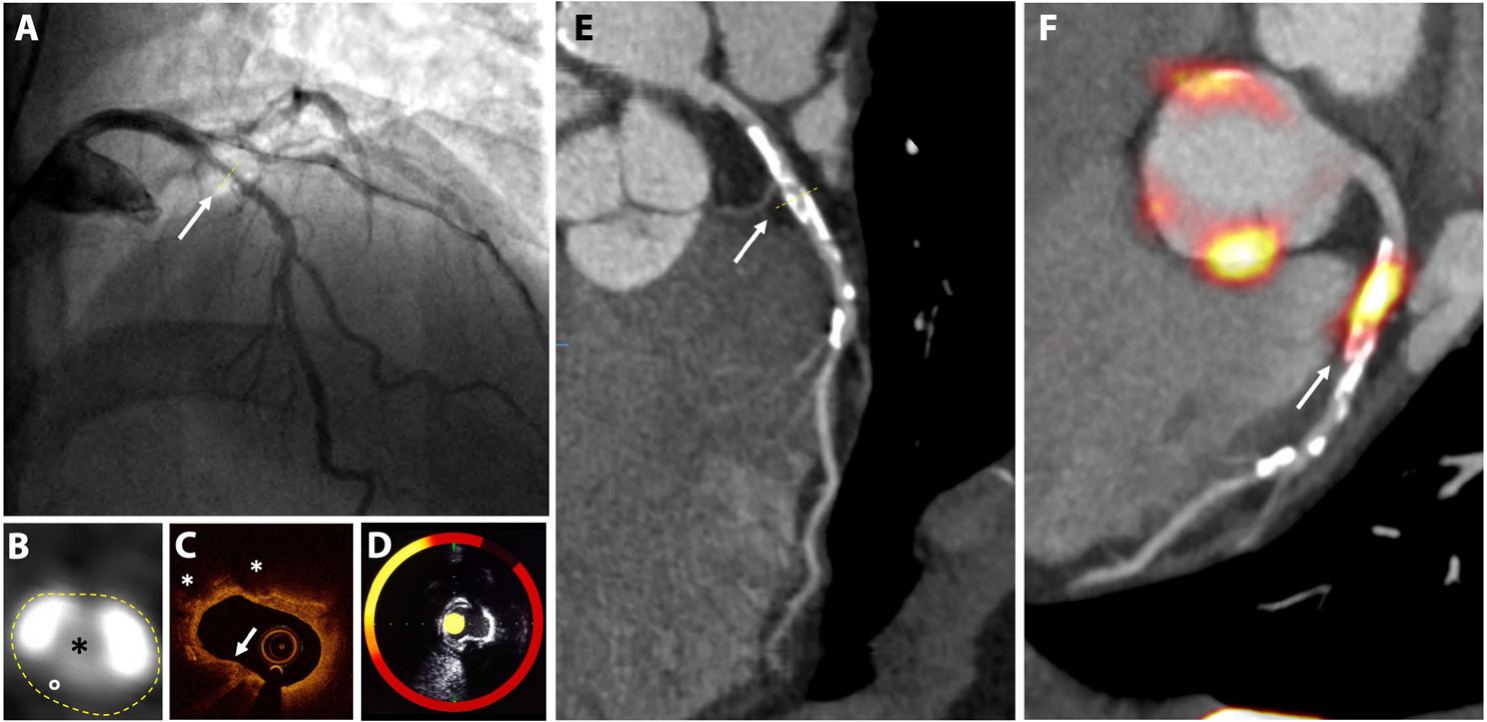
Imaging of coronary atherosclerosis in a patient with non-ST elevated myocardial infarction. (A) Catheter angiography shows an irregular lesion in the proximal left anterior descending coronary artery (artery). (B) Transaxial view of the coronary lesion on CT (i) shows a complex plaque with calcified (white) and fibrofatty (°) plaque around a central lumen (*). (C) Optical coherence tomography detects a thin fibrous cap (arrow) and lipid pools (*). (D) Combined near infrared spectroscopy and intravascular ultrasound confirms high lipid burden within the plaque (yellow). (E) Centreline reconstruction of the left anterior descending artery visualizes calcification and plaque formation throughout the entire vessel. (F) ^18^F-Sodium fluoride positron emission tomography/ CT detects high uptake in the atherosclerotic plaque

Optical coherence tomography (OCT) provides exceptionally detailed images of the fibrous cap using near infrared light delivered via a fibre optic wire (Figure 2C). The OCT catheter is positioned over a guidewire and a blood-free pool is created by injecting saline or contrast media. Using fast frequency domain analysis, OCT captures detailed images of the adjacent thin fibrous cap. The findings correlate well with histological features.^15^ High signals in the fibrous cap signify macrophage adherence and thrombus formation.^16^ These high resolution benefits need to be balanced against reduced tissue penetration which limits assessment of deeper plaque constituents. OCT is used clinically to detect vulnerable plaques and guide therapy in the coronary arteries.^17,18^ OCT is now being used for these purposes in carotid artery stenosis^19^ and peripheral vascular disease.^20^

#### Near infrared spectroscopy/intravascular ultrasound

Near infrared spectroscopy (NIRS) is another catheter-based invasive technique. NIRS does not require a blood free field and uses wave scatter to produce a gradient map corresponding to the probability of adjacent lipid (Figure 2D). The resultant lipid-core burden index (LCBI) describes the ratio of high lipid content in adjacent structures against the total study area. Modern probes are combined with intravascular ultrasound (IVUS) to provide structural context to the morphological data.^21^

IVUS is a more established and, consequently, cost-effective technique than OCT and NIRS. It packages a high-frequency ultrasound probe in a catheter that can directly visualize adjacent atherosclerotic plaque from within the lumen (Figure 2D). High-resolution greyscale images show the structure of atherosclerotic plaque and the adjacent vessel wall. Features of plaque components can be identified by analyzing the backscatter and can reliably reveal the lipid-rich necrotic core, calcification and fibrofatty plaque.^22,23^ Despite reasonable tissue penetration, IVUS lacks the spatial resolution to measure the thickness of the fibrous cap. Like conventional ultrasound, mineralized calcium deposits cast acoustic shadows, thereby obscuring underlying tissue detail.

NIRS/IVUS is well established in detecting and treating coronary disease.^15,24^ Its use has been validated to perform a similar function in the carotid^25^ and lower limb^26^ arteries.

### Non-invasive imaging

#### Ultrasound

In superficial vessels, such as the carotid and limb arteries, duplex ultrasound combines structural and functional data to quantify the degree of stenosis caused by atherosclerotic plaque. The ratio of peak systolic velocity proximal and distal to suspected lesions is used to estimate the degree of stenosis. Ultrasound can be used to measure the total plaque area^27^ and the greyscale pixel intensity of atherosclerotic plaque images have been shown to correspond with histological features.^28^ Ultrasound is non-invasive, radiation-free and portable. Hence, it is often used as the first-line imaging modality to quantify stenoses throughout the peripheral vascular tree.

The high resolution of ultrasound can differentiate components of the arterial wall in superficial vessels. Pathological proliferation of the intimal-medial layer is a sign of early subclinical plaque formation.^29^ Detecting this thickening using ultrasound can be used as a "window" to an individual’s global cardiovascular health. Indeed, increased IMT is associated with significantly increased risk of myocardial infarction, stroke and death.^30^

Contrast agents containing a homogenous suspension of inert gas microbubbles (*e.g.* SF_6_) administered in the venous space can highlight specific features of arterial atherosclerotic plaque. In the carotid arteries, microbubbles identify neovascularization in culprit lesions with a sensitivity and specificity greater than 80%.^31^ In patients with abdominal aortic aneurysms, contrast-enhanced ultrasound provides real-time characterization of luminal flow. Visualization of complications following endovascular repair, such as endoleaks, is feasible.^19,32^ The lack of ionizing radiation makes contrast-enhanced ultrasound an ideal modality for situations that require repetitive imaging.

Standardizing the ultrasound assessment of vascular beds reduce the risk of interobserver variability. Vessel assessment is not always possible if the view is obscured by densely calcifying atherosclerotic disease. Similarly, bone or gas overlying the target vessel prevents adequate visualization using ultrasound, limiting its use to easily arteries that are easily accessible.

#### Computed tomography

The use of CT angiography (CTA) is well established to assess the cardiovascular system. It is non-invasive and accessible. CTA has the added benefit of visualizing the entire vessel from its origin to target structure, even in tortuous vessels. Among its many applications, CTA has the spatial resolution to detect focal luminal stenoses and provide a global assessment of vascular disease. Owing to the short acquisition times of new generation CT scanners, it is now possible to image the coronary vessels in great detail. Indeed, CTA is highly accurate in determining coronary stenosis severity and has been incorporated within many clinical guidelines as the first-line imaging modality for individuals with suspected cardiac chest pain. CTA is used throughout the vascular tree. In the carotid arteries, CTA has a reported sensitivity approaching 100% and specificity of 63% (95% confidence interval 25–88%) to detect a stenosis greater than 70%.^22,33,34^

Beyond the assessment of luminal stenosis, CT can characterize plaque morphology. Several high-risk plaque features such as positive remodelling, spotty calcification, high-attenuation fibrous plaques and low-attenuation lipid-rich necrotic plaques can be identified.^35–37^ The napkin-ring sign on centreline reconstruction of the coronary vessels represents differentiation between the fibrous plaque and necrotic core.^38^ It is characterized by a crescentic high attenuation pattern surrounding a low-attenuation atheromatous lesion distinct from the vessel lumen—a morphological pattern that mirrors plaque histology.

CT is particularly well suited to visualize vessel calcification. However, beam hardening in relation to densely calcified plaque causes blooming that exaggerates plaque size and obscure the lumen*—*particularly in small calibre vessels with a high burden of calcification. In acute disease, CT is unable to differentiate soft-tissue components of a plaque. For instance, it is not possible to distinguish directly a stable fibroatheromatous lesion from acute plaque haemorrhage or thrombosis. Identification of the culprit plaque relies on a combination of radiological features along with the clinical presentation and pattern of end-organ damage.

#### Magnetic resonance imaging

Angiography using MRI is best suited to imaging large stable vessels such as the carotid arteries. Multicontrast MRI (*T*_1_ weighted, *T*_2_ weighted, proton density) offers excellent soft tissue characterization. This allows the constituents of atherosclerotic plaque to be investigated without the need for ionizing radiation. These properties make MRI useful in the longitudinal studies of chronic cardiovascular diseases. Administration of gadolinium (Gd)-based contrast media improves image acquisition times and provides further structural information outlining differences between the blood pool and vessel wall.^39^

Motion artefact near the heart makes imaging small vessels challenging. Advances in MRI have addressed this limitation. Accelerated image acquisition allows for a reduction of motion artefacts and noise.^40^ Bright-blood techniques use blood itself as an intrinsic contrast agent, thus reducing the requirement of gadolinium-based contrast agents. Now, a 1.5 T MRI machine can detect significant left main stem or three-vessel coronary disease in up to 94% of patients.^41^ Whilst image quality remains inferior to CT coronary angiogram, MRA can be useful in the assessment of coronary aneurysms and aberrant coronary ostia.^42^ Additionally *T*_1_ weighted imaging can be applied to the coronary arteries for the detection of intraluminal thrombus or intra plaque haemorrhage. MRI has the extra benefit of assessing cardiac dynamics along with myocardial perfusion and viability. Ongoing advances in cardiac MRI make it a promising imaging modality of the future.

In the carotid arteries, the superior soft tissue discrimination of MRI allows the measurement of the fibrous cap thickness and visualization of the necrotic core.^43^ The sensitivity to detect lipid-rich cores can be further improved using Gd-based contrast media.^44^ Following an acute event, such as a transient ischaemic attack from an internal carotid artery plaque rupture, *T*_1_ weighed MRI can detect intraplaque haemorrhage and thrombus. This correlates with the risk of future ischaemic events.^45,46^ Higher field strengths reduce background noise and artefact. Calcium typically appears hypodense, whereas fibrous tissue has a low signal intensity on *T*_1_ and high signal intensity on *T*_2_ weighted images.

##### “Smart” Contrast Agent MRI

**Figure 3.**
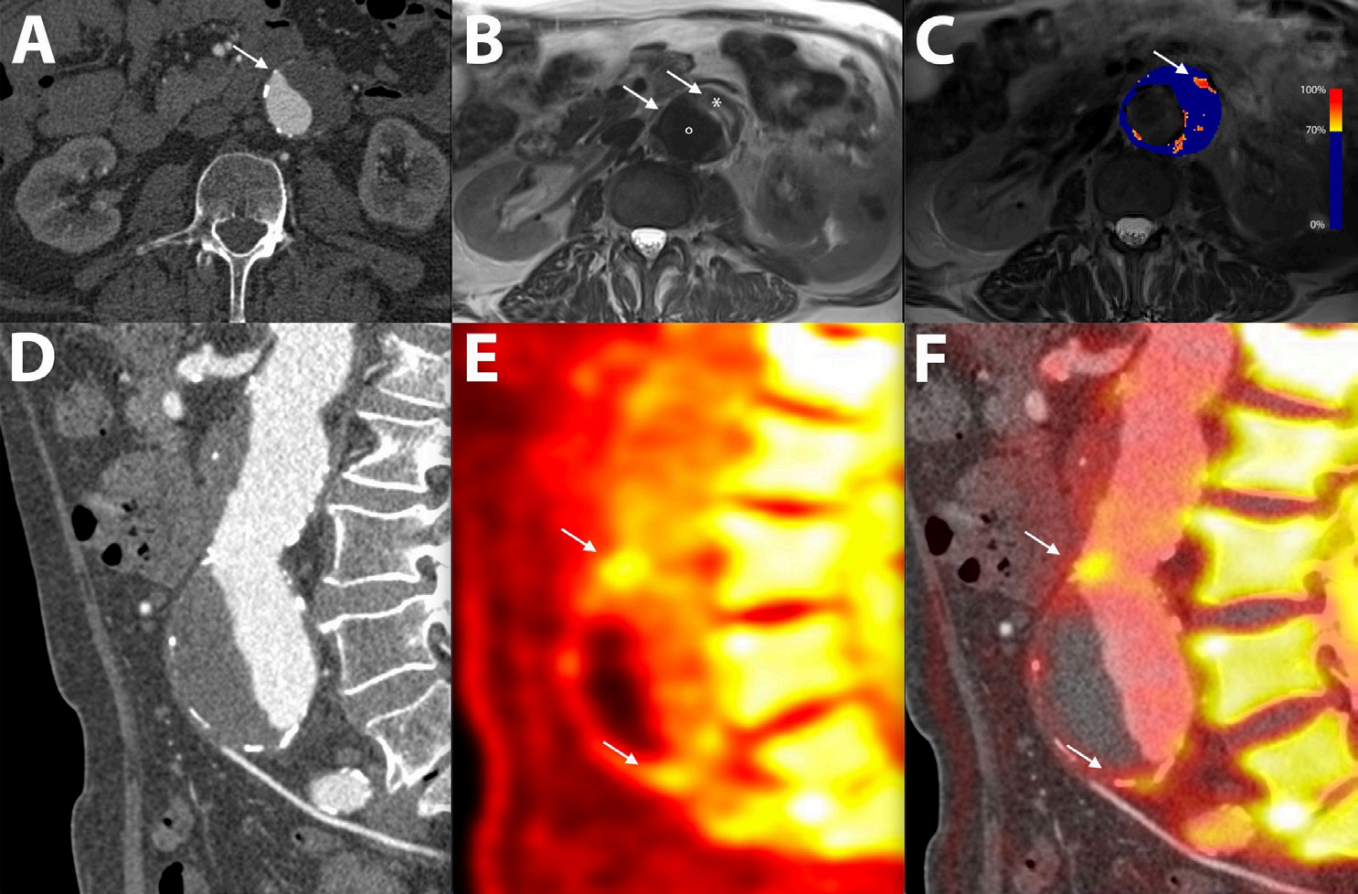
CT, MRI and PET in a patient with a juxtarenal abdominal aortic aneurysm. (A) Transverse view of the aneurysm as seen on CT shows a dilated aorta with thrombus. (B) *T*_2_ weighted MRI of the same aorta differentiates between the lumen (•), thrombus (*) and adjacent structures. (C) A parametric map of the difference in *T*_2_* MRI intensity before and after the administration of ultrasmall particles of iron oxide uptake shows high focal uptake in the anterior wall of the aneurysm (arrow). (D) The sagittal CT view delineates the morphology of the aneurysm. (E) ^18^F-Sodium fluoride PET shows uptake within the anterior aortic wall (arrows) detects areas of greatest vascular injury. (F) Superimposing PET over the CT confirms high ^18^F-Sodium fluoride uptake at the aneurysm neck and near the bifurcation (arrows). PET,positron emission tomography

Ultrasmall paramagnetic particles of iron oxide (USPIOs) are 30 nm iron oxide nanoparticles stabilized with low-molecular-weight dextran. USPIOs accumulate in macrophages following phagocytosis and remain in the circulation for extended periods.^47^ Areas rich in USPIO-positive macrophages have a low-signal intensity on *T*_2_ and *T*_2_* weighted MRI.^48^ In the carotid arteries, USPIO accumulation within atherosclerotic lesions coincides with active plaque disease. These plaque exhibit intense macrophage infiltration.^49^ USPIO also accumulate with high affinity in areas of macrophage infiltration within abdominal aortic aneurysms (Figure 3C).^50^ Using smart MRI contrast agents to detect cellular activity within the vascular bed carries immense promise. These techniques may ultimately lead to the detection of active plaques at risk of imminent events and allow preventative therapy.

##### Magnetic Resonance Spectroscopy

MR spectroscopy (MRS) combines the spatial imaging obtained from MRI with spectral analysis to detect the chemical composition and metabolic state of cardiovascular tissue. MRS is able to detect a range of atoms, including 1-Hydrogen (^1^H), 31-Phosphorus (^31^P), and 13-Carbon (^13^C).^51^*In vivo* carotid studies using MRS have successfully quantified cholesteryl esters within atherosclerotic plaque.^46,52^ Cholesteryl esters are the major class of lipids found in the lipid-rich necrotic core of vulnerable plaques. The chemical composition of structures is obtained using a chemical shift imaging sequence to acquire spectra over and around the atherosclerotic plaque. MRS amplitudes for specific metabolites, such as lipids, are then interpreted as a ratio to the amplitude of intrinsic water.^53,54^ The final analysis allows detection and quantification of the lipid content of atherosclerotic plaque.

## Molecular imaging

Biologically active atherosclerotic lesions are inherently unstable and prone to rupture.^55^ Targeted biological tracers enable positron emission tomography (PET) and single proton emission computed tomography (SPECT) to detect increased activity of specific disease processes, such as increased glycolytic activity or microcalcification.

Biological radiotracer molecules typically consist of two components. One section has a ligand that targets sites of specific disease activity. The other component consists of a radioisotope. PET and SPECT scanners can detect the intensity and distribution of radiotracer activity following molecular engagement with target disease processes. PET has superior image definition and has gained popularity over SPECT.

Molecular imaging in cardiovascular medicine has made substantial advances recently with an ever-expanding array of biological tracers targeting different processes. Approaches to standardize quantification of radiotracer uptake has improved the reporting and reproducibility of results.^56^

### Glucose and glycolysis

^18^F-Fluorodeoxyglucose (^18^F-FDG) is a glucose analogue. It is the most commonly used biological tracer in clinical practice. ^18^F-FDG is taken up by metabolically active cells and its immediate metabolite is trapped within the cell following phosphorylation. This allows the quantification of cellular glycolytic activity. In vascular inflammation, uptake of ^18^F-FDG is pronounced in smooth muscle cells, endothelial cells and macrophages.^57^ Increased ^18^F-FDG uptake in carotid and coronary atherosclerosis correlates with unstable plaques and those that exhibit histological features of vulnerability,^58,59^ including increased CD68 macrophage density.^60^

Since ^18^F-FDG reflects glycolytic activity, uptake is non-specific. This limits interpretation of ^18^F-FDG PET in structures adjacent to those with a high physiological affinity to glucose, such as the myocardium. In order to visualize ^18^F-FDG PET uptake in the coronary arteries, it is necessary to suppress myocardial uptake using a low-carbohydrate, high-fat preparatory diet. However, these attempts at myocardial suppression are ineffective in around a quarter of patients, rendering assessment of coronary plaque activity very challenging.^61^

### Macrophages and inflammation

Tracers that target macrophages directly, such as the somatostatin subtype 2 (SST2) receptor analogues, overcome the limitations caused by the poor specificity of ^18^F-FDG. Here, the combination of a somatostatin ligand with a DOTA- (1,4,7,10-Tetraazacyclododecane-1,4,7,10-tetraacetic acid) or NOTA- (1,4,7-Tricarboxymethyl-1,4,7-triazacyclononane) based “cage” has resulted in the development of various tracers. The cage houses a positron-emitting isotope, such as Gallium-68 (^68^Ga) or Copper-64 (^64^Cu). PET studies reveal that these agents exhibit preferential vascular uptake in patients with established cardiovascular risk factors and adverse Framingham risk scores.^62^

Histological comparison of carotid plaque with high^63^ Ga-DOTA-TATE (1,4,7,10-tetraazacyclododecane-N,N',N'',N'''-tetraacetic acid]-D-Phe,^1^ Tyr^3^-octreotate) uptake reveals selective binding of the radiotracer to CD68-positive macrophage-rich lesions. In a pilot study,^63^Ga-DOTA-TATE correctly identified metabolically active coronary and carotid lesions with good reproducibility and higher sensitivity than ^18^F-FDG.^64^

Other tracers targeting macrophage activity include vascular cell adhesion molecule-1 (VCAM-1), ^11^C-choline and ^18^F-fluthymidine. Chief amongst these are translocator protein (TSPO) ligands (^11^C-PK11195), which are 18 kDa proteins expressed in the mitochondria of most cells. Activated macrophages exhibit marked upregulation of these translocator proteins. Preliminary studies suggest that ^11^C-PK11195 PET/CT correctly identifies suspected carotid lesions and has favourable tissue-to-background ratios compared to ^18^F-FDG.^65^

Activated inflammatory cells within atherosclerotic plaque exhibit CXCR-4 receptors. Novel PET radiotracers, such as^63^Ga-Pentaxifor, can be used to target these receptors.^66^ In the coronary arteries,^63^Ga-Pentaxifor is high in culprit vessels following acute myocardial infarction (median maximum standardized uptake value 1.96, interquartile range 1.55–2.31). These lesions also have high concentrations of CD68 +macrophages.^67^

### Microcalcification

^18^F-Sodium fluoride (^18^F-NaF) binds to exposed hydroxyapatite crystals. Due to surface area effects, it preferentially binds to areas of developing microcalcification, which is beyond the resolution of CT.^63,68^ Because ^18^F-NaF is not taken up by myocardium, the background signal is low, which allows even relatively low signal to be visualized within the coronary vessels (Figure 2)*—*a significant advantage over ^18^F-FDG.

*In vivo* studies have demonstrated binding of ^18^F-NaF to areas of active calcification which are associated with plaque instability. Prospective studies have established high ^18^F-NaF uptake in culprit coronary^69^ and carotid plaques.^69^ In carotid plaque, the findings correlated strongly with histological features of high-risk plaques, including macrophage infiltration, necrosis and apoptosis. In culprit coronary vessels, high ^18^F-NaF uptake shows good agreement with high-risk features on intravascular ultrasound.^69^

The application of ^18^F-NaF PET/CT extends to vascular calcification in the wider arterial tree. In a prospective study of 72 patients with abdominal aortic aneurysms, there was marked uptake of ^18^F-NaF within the wall of aneurysms compared to non-aneurysmal sections and the aorta of healthy controls. High ^18^F-NaF uptake suggested weakening of the aortic wall and greater aneurysm expansion or rupture.^70^ These findings draw a link between metabolically active abdominal aortic aneurysms and morphological disease progression.

### Hypoxia and angiogenesis

^18^F-Flumisonidazole (^18^F-MISO) is a biological tracer that concentrates in hypoxic viable cells due to an accumulation of its metabolites in an oxygen-deprived environment. Animal models show ^18^F-MISO correlates well with aortic atherosclerosis and areas of FDG uptake.^71^ Exploratory human studies suggest that ^18^F-MISO PET/CT corresponds to areas of vessel hypoxia, increased macrophage density and ^18^F-FDG uptake in symptomatic carotid artery disease.^68,72^

Angiogenic endothelial cells and hypoxic macrophages in atherosclerotic plaque express α_v_β_3_ integrin cell surface glycoproteins. In animal models, α_v_β_3_ targeted imaging with MRI sensitive paramagnetic particles identify areas of angiogenic proliferation.^73^ A novel PET tracer, ^18^F-Galacto-RGD, can also target α_v_β_3_ integrin^74^ and in mice models of atherosclerosis, binds to sites of new atherosclerotic plaque. However, histological analysis in these mice models showed that uptake co-localized in macrophage-rich atherosclerotic plaque, as opposed to angiogenesis specifically.^75^ Culprit carotid plaques demonstrate increased ^18^F-Galacto-RGD uptake on pre-operative PET/CT and post-operative autoradiography analysis. In a small sample of patients, ^18^F-Galacto-RGD binding showed a tendency to bind to plaque rich in macrophages and those with increased vasa vasorum density.^76^

## Clinical translation

Anatomical imaging is part of routine clinical care. Advances in cardiovascular imaging now allow plaque characterization over and above the degree of vessel stenosis. Multimodality techniques enable *in vivo* detection of high-risk features and culprit plaques (Table 1). Biological PET tracers can detect and quantify specific disease processes before they manifest as structural changes or cause clinically significant events. Improved diagnosis, accurate risk prediction and targeted treatment remain a crucial objective in the management of atherosclerotic conditions.

**Table 1.** Imaging modalities to detect high-risk features of atherosclerotic plaque

High risk plaque feature	Optical imaging	Ultrasound scan	CT	MRI	PET
Vessel stenosis or occlusion	OCT	Duplex ultrasound scan	CT angiogram	MR angiogram	-
Thin fibrous cap	OCT	-	-	-	-
Large necrotic core	OCT, NIRS	IVUS, virtual histology	Centre-line arterial reconstruction	*T_1_* weighted high intensity plaque imaging	-
Angiogenesis and intraplaque haemorrhage	OCT	Duplex ultrasound scan, IVUS, contrast enhanced ultrasound	-	*T_1_* weighted high intensity plaque imaging, α_v_β_3_ targeted paramagnetic particles	^18^F-MISO, ^18^F-Galacto-RGD
Subclinical plaque rupture	OCT, NIRS	Duplex ultrasound scan, IVUS	-	*T_1_* weighted high intensity plaque imaging	Novel fibrin and platelet targeted biotracers
Glycolytic activity	-	-	-	-	^18^F-FDG
Macrophage infiltration	OCT	-	-	USPIO	^18^F-DOTATATE, VCAM-1, ^11^C-Choline, ^18^F-Choline,^11^C-PK11195
Microcalcification	-	-	-	-	^18^F-Sodium Fluoride (NaF)

IVUS, intravascular ultrasound; NIRS, near infrared spectroscopy; OCT, optical coherence tomography; PET, positron emission tomography; USPIO, ultrasmall paramagnetic particles of iron oxide.

### Coronary artery disease

The clinical presentation of occlusive coronary disease is variable. Contemporary imaging of the coronary vessels addresses two important objectives*—*the assessment of total plaque burden and the detection of end-organ ischaemia. Complementary imaging techniques can inform on the functional and metabolic state of atherosclerotic plaque. This adds a new perspective to plaque assessment beyond established imaging techniques.

The assessment of patients with stable chest pain highlights the evolving role of imaging to aid diagnosis. Traditionally, the risk of coronary artery disease was estimated using clinical risk prediction tools. If a clinical diagnosis was suspected, exercise testing and myocardial perfusion studies were recommended. NICE amended its guidelines in 2016 to reflect the benefits offered by CT coronary angiography (CTCA) and now recommends CTCA as the first-line investigation in patients with stable chest pain of suspected cardiac origin. The SCOT-HEART trial showed that, in patients with stable chest pain, CTCA reclassifies the diagnosis of coronary artery disease in up to a quarter of patients and improves the accuracy of diagnosing angina.^77^ Indeed, the sensitivity and negative predictive value of CTCA is 99% when using a cut-off of 50% stenosis.^78^ Hence, CTCA offers a reliable and low-risk approach to exclude significant coronary artery disease in low- and medium-risk groups.

Coronary stenosis alone does not predict the risk of future cardiovascular events. Instead, events appear most closely related to adverse plaque features and total burden: the more plaque you have, the more likely one will rupture and cause a myocardial ischaemia.^79^ The coronary artery calcium (CAC) score is a non-contrast CT technique that estimates overall calcification in vascular beds. The CAC score provides an improvement in predicting future cardiovascular events in patients at medium risk of ischaemic heart disease (Framingham risk score >10%).^80^ In particular, CAC scores capture patients at high risk, who would otherwise be incorrectly classified using traditional scoring methods.^81^

Multimodality imaging adds a further dimension to the assessment of patients with chest pain. For instance, in patients with acute coronary syndrome, the lipid core burden index on NIRS independently predicts future cardiovascular events (Hazard ratio 1.19, 95% confidence ratio 1.07–1.32, *p* = 0.001).^82^ In the heart, PET has traditionally remained a challenging area due to the non-specific uptake of ^18^F-FDG by the surrounding myocardium.^69^ However, uptake of ^18^F-NaF is more selective to regions of microcalcification. This makes it an ideal agent for studying the coronary arteries. In a study of 80 patients, ^18^F-NaF preferentially bound to IVUS-confirmed culprit coronary plaques.^69^ Similarly, a study of 119 participants showed that ^18^F-NaF PET/CT findings correlated with underlying angina (*p* = 0.023), prior cardiovascular events (*p* = 0.016) and Framingham risk scores (*p* = 0.011).^83^ The predictive power of ^18^F-NaF to identify culprit coronary plaques is the focus of an ongoing prospective multicentre trial in patients with recent acute coronary syndrome and multivessel disease: the PRE^18^FFIR study (NCT02278211). Whether this can be enhanced by other novel radiotracers, such as ^68^Ga-DOTATATE,^84^ remains to be established.

Non-invasive multimodality imaging enables the detection of high risk features *in vivo* and promises improved risk prediction. Such high-risk groups could be targeted for more intensive prophylactic therapy, such as the emerging proprotein convertase subtilisin/kexin Type 9 (PCSK9) inhibitors. However, the added value of such imaging will need to be established in prospective trials, such as the DIAMOND study (NCT02110303) where the benefit of dual antiplatelet therapy in patients with high coronary ^18^F-NaF PET uptake is being assessed.

#### Carotid artery disease

Extracranial carotid plaque rupture results in acute thrombus formation that may occlude the internal carotid artery or be the lead point for dissection. The thrombus may embolize to the brain or the eye causing permanent tissue loss. The goal in treating carotid artery disease is to prevent future thromboembolic events. Imaging the carotid arteries fulfils an important role in cerebrovascular risk prediction. The NASCET^85^ and ECST^86^ studies showed the benefit of endarterectomy in symptomatic high-grade carotid artery stenosis.^87^ Ultrasound is the mainstay of risk stratification of symptomatic carotid artery disease.^88^

We now know that early surgical intervention following an index carotid artery event is important to reduce recurrent embolic episodes from carotid artery lesions. Patients may have tandem disease. This group was excluded from the original NASCET trials. Hence, many guidelines do not advocate duplex ultrasound as the sole imaging arbiter prior to surgery. CT and MR angiography have the benefit of overcoming vessel tortuosity and visualizing the entire artery.^89^ However, nearly a third of recurrent disabling or fatal strokes occur in patients with internal carotid artery stenosis less than 50%.^86^ Ultrasound, CT and MR are limited to studying the morphological features of atherosclerotic plaque. They cannot reliably predict future cerebrovascular events in “low” risk groups with lesser degrees of stenosis.^89^

**Figure 4.**
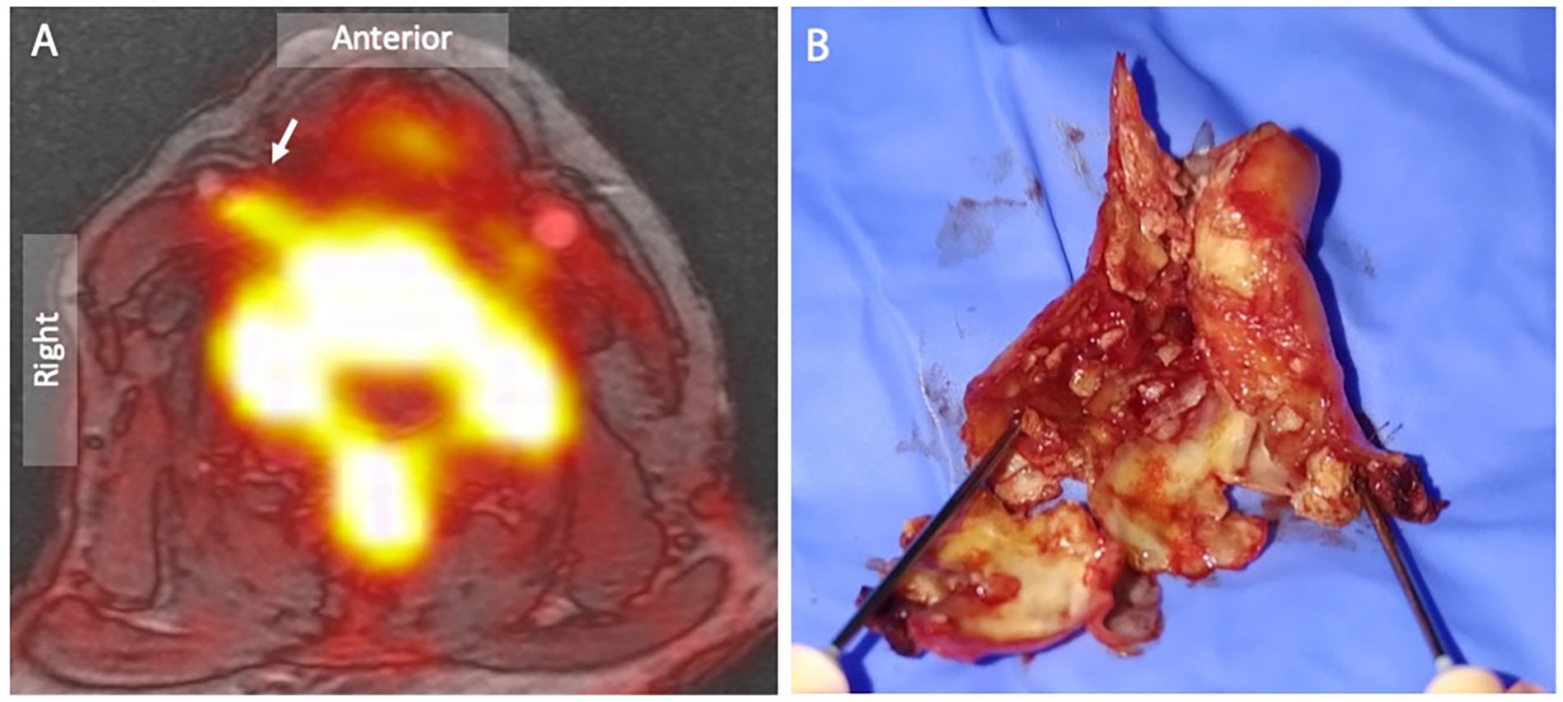
^18^F-Sodium fluoride positron emission tomography and magnetic resonance angiography of a symptomatic right internal carotid artery lesion. (A) Combined ^18^F-Sodium fluoride positron emission tomography superimposed on MR angiogram localizes focal radiotracer uptake in the culprit right internal carotid artery plaque (arrow). (B) Surgical endarterectomy confirms a highly ulcerated lesion with positive remodelling and marked intimal irregularity

Additional imaging techniques, such as contrast-enhanced ultrasound, and multiparametric or USPIO-enhanced MRI, can identify unstable and culprit atherosclerotic plaque. Moreover, PET imaging with ^18^F-FDG or ^18^F-NaF PET can detect the biological activity of culprit plaques, with ^18^F-NaF in particular binding to culprit carotid lesions characterized by a lipid-rich necrotic core (Figure 4).^69^ The combination of PET/MRI offers a substantial improvement with the potential to assess simultaneously for flow, thrombus, lipid core, plaque rupture and necrotic unstable plaque with microcalcification.

The treatment of asymptomatic patients with carotid artery disease remains controversial.^90^ Similarly, there is no consensus on how to best treat symptomatic patients with internal carotid artery disease below the current threshold for intervention. Multimodal imaging has the potential to deliver this better risk prediction in a non-invasive and powerful manner. The SAFFIRE studies (NCT03215563 and NCT03215550) will assess the clinical significance of ^18^F-NaF PET/MRI in relation to plaque vulnerability, the requirement for surgery and clinical outcomes. This may lead to better risk stratification of patients with stroke permitting better selection for surgery: avoiding unnecessary carotid endarterectectomy whilst encouraging intervention in high-risk plaque below the current threshold for surgical intervention.

#### Aortopathy

The pathobiology of abdominal aortic aneurysms shares key features with atherosclerotic disease. Ultrasound scan remains the cornerstone of abdominal aortic aneurysm screening and surveillance. However, current prediction models rely on a single morphological measurement of anteroposterior diameter to prognosticate the risk of aneurysm rupture. In males, this diameter is 55 mm^91^ although many advocate a lower threshold for females. Endovascular or open surgical repair is considered once aneurysms reach this threshold.^92^

Some aneurysms may rupture at sizes smaller than 55 mm whilst others reach large dimensions without causing any harm. Like carotid artery disease, multimodality imaging may provide better risk stratification than ultrasound scan-derived diameter alone.

MRI signal intensity can characterize thrombus organization with high accuracy. A high *T*_1_-signal is seen in disorganized thrombus that is typically unstable.^93^ A series of 35 patients with abdominal aortic aneurysms found that individuals with high thrombus *T*_1_ signal intensity were likely to exhibit twice as fast aneurysm growth compared to those with organized thrombus.^94^ It is uncertain whether thrombus formation accelerates aneurysm growth. One possible explanation is that organized thrombus is the product of chronic stable aneurysm morphology, whereas a rapidly growing aneurysm has a large turnover of thrombus that is inherently unstable.

Smart contrast agents such as USPIO can further characterize biological activity within the aneurysm wall. High aortic mural USPIO uptake localizes to areas of macrophage infiltration and reflects vessel wall inflammation. An increased burden of USPIO uptake is observed in aneurysms with faster rates of expansion.^48^ In aneurysmal disease, ^18^F-NaF PET/CT detects aortic microcalcification. This is a common end point of multiple pathological processes ultimately leading to aortic degeneration. Increased ^18^F-NaF uptake is associated with faster aneurysm growth and an increased risk of rupture or requiring repair, independent of aneurysm diameter.^70^

Timely intervention is invaluable prior to the catastrophic consequences of aneurysm rupture. Conversely, better risk prediction may aid the decision of conservative aneurysm management in patients with major comorbidities. Much like carotid disease, multimodal imaging may provide useful insights into future risk and be a better guide for surgical intervention in patients with aneurysms above and below the current standard of care to consider intervention at a binary cut point of aneurysm diameter above 55 mm.

#### Peripheral vascular disease

Peripheral vascular disease is a heterogenous group of occlusive arterial conditions involving the lower limbs. Atherosclerosis primarily causes intimal calcification. In contrast, diabetes mellitus and chronic kidney disease cause arterial stiffness by enhancing transmural calcification of the medial layer of the artery. Both processes are initiated by microcalcification.

Arterial duplex, MR and CT angiogram can visualize the lumen in large vessels but may either over estimate or underestimate the degree of stenosis in narrower arteries, such as below the knee. For instance, calcification in these small vessels causes a blooming artefact in CT Angiograms that obscures the view of the lumen. When calcification is not present, a contrast-enhanced residual lumen can be over estimated in CT with voxels that are too large. Hence, diagnostic angiography remains the gold-standard to visualize luminal narrowing in the lower limbs owing to its high spatial resolution. This approach, however, does not image the arterial wall or inform on the biological activity within it.

^18^F-Fluoride binds with good affinity to femoral plaque and is better at detecting inflammation compared to ^18^F-FDG.^95^ A study of 409 oncology patients undergoing ^18^F-NaF PET/CT were incidentally found to have significant femoral artery ^18^F-fluoride uptake. The degree of uptake correlated strongly with hypertension, hypercholesterolaemia, smoking habit, diabetes mellitus and prior cardiovascular events.^96^ The clinical progression of this uptake in longitudinal studies remains to be validated.

#### Drug evaluation

Multimodal imaging can provide objective surrogate end points to confirm target engagement and treatment efficacy in relatively modest-sized populations.

One example is the study of statins in cardiovascular disease. The effect of statins on atherosclerotic burden has been extensively investigated using invasive^97–99^ and non-invasive^100–102^ imaging techniques. Intravascular imaging using OCT and IVUS was used to study the effects of statins on atheroma in trials such as ASTEROID,^97^ ESTABLISH^99^ and REVERSAL.^103^ Similarly, MRI has been used to quantify the reduction in the lipid content of necrotic core after treatment with rosuvastatin at 1 and 2 years of follow-up.^100,101^ The ATHEROMA trial used USPIO-enhanced MRI to show a reduction in carotid plaque inflammation within 3 months of starting atorvastatin.^102^

Imaging may be used to validate the effect of therapeutic interventions. The REMNANT trial studied the effect of thrombus aspiration from culprit plaques in patients with acute coronary syndrome. The investigators used IVUS to quantify the percentage increase in lumen volume following thrombus aspiration and showed that aggressive intervention was likely to result in successful stent deployment.^104^ Conversely, MRI and PET/CT have been used to determine a lack of clinical efficacy. The dal-PLAQUE study did not show a reduction in vessel wall inflammation using the CTEP inhibitor, dalcetrapib: an agent that is now known to be clinically ineffective.^105^

## Conclusion

Detailed structural and functional assessment of the vascular tree is possible with conventional imaging modalities. However, the promise of identifying vulnerable atherosclerotic plaque leading to cardiovascular events remains elusive. Multimodality cardiovascular imaging provides the clinician with valuable information on the morphology and metabolic state of atherosclerotic diseases. Combining imaging modalities offers powerful tools to study specific disease processes in the context of diagnosis, risk prediction and targeted intervention.

A paradigm shift to move away from the assessment of stenosis severity alone is required. Detecting high-risk plaque features promises to reclassify low- and medium-risk individuals who are, in fact, at high risk of developing adverse cardiovascular events. Correctly identifying this group is the greatest unmet clinical need in contemporary cardiovascular imaging. Incorporating novel imaging techniques addresses the limitations of modern stenosis-driven approaches. Translating these wide range of imaging techniques into clinical practice is the next major advance in atherosclerotic plaque assessment. Ongoing research on the role of metabolic imaging promises to detect and prognosticate cardiovascular disease progression. These studies will better inform the translation of novel imaging modalities to mainstream clinical practice.
